# Risky Alcohol Consumption and Associated Health Behaviour Among HIV-Positive and HIV-Negative Patients in a UK Sexual Health and HIV Clinic: A Cross-Sectional Questionnaire Study

**DOI:** 10.1007/s10461-019-02714-2

**Published:** 2019-10-29

**Authors:** Emmi Suonpera, Rebecca Matthews, Ana Milinkovic, Alejandro Arenas-Pinto

**Affiliations:** 1 Centre for Clinical Research in Infection and Sexual Health, UCL Institute for Global Health, University College London, London, UK; 2 The Mortimer Market Centre, Central and North West London NHS Foundation Trust, London, UK; 3 Chelsea & Westminster Hospital NHS Foundation Trust, London, UK; 4 MRC Clinical Trials Unit at UCL, London, UK; 5 Mortimer Market Centre, UCL Institute for Global Health, Off Capper Street, London, WC1E 6JB UK

**Keywords:** HIV, Alcohol misuse, AUDIT, Drug use, Mood disorders

## Abstract

Alcohol misuse has been associated with negative consequences among HIV-positive patients. Data on real prevalence of risky alcohol consumption among the HIV-positive population in the UK are lacking. A cross-sectional questionnaire study using standardised validated instruments among HIV-positive (n = 227) and HIV-negative (n = 69) patients was performed. The prevalence of risky alcohol consumption (AUDIT) and associations with depressive symptoms (PHQ-9), problematic drug use (DUDIT), adherence to ART (CASE Adherence Index), sexual behaviour and demographic characteristics were assessed among both patient groups independently. A quarter (25.1%) of HIV-positive patients and 36.1% of HIV-negative patients reported risky alcohol consumption (AUDIT-score ≥ 8). In the multivariable analysis among HIV-positive patients depressive symptoms (p = 0.03) and problematic drug use (p = 0.007) were associated with risky alcohol consumption. Among HIV-negative patients these associations were not present. Risky alcohol consumption among HIV-positive patients is prevalent, and together with depressive symptoms and problematic drug use, may influence HIV-disease progression and patients’ wellbeing.

**Electronic supplementary material:**

The online version of this article (10.1007/s10461-019-02714-2) contains supplementary material, which is available to authorized users.

## Resumen

El abuso del alcohol se ha asociado con consecuencias negativas a la salud de pacientes VIH-positivos. La prevalencia actual del consumo riesgoso de alcohol en la población VIH-positiva en el Reino Unido es desconocida. Este estudio transversal, basado en un cuestionario y usando instrumentos validados y estandarizados, incluyó pacientes VIH-positivos (n = 227) y VIH-negativos (n = 69). La prevalencia del consumo riesgoso de alcohol (AUDIT) y su asociación con síntomas de depresión (PHQ-9), uso problemático de drogas (DUDIT), adherencia a la TAR (CASE Adhernce Index), comportamiento sexual características demográficas que fueron evaluadas independientemente en cada grupo de pacientes. La cuarta parte (25.1%) de los pacientes VIH-positivos y 36.1% de los VIH-negativos reportaron consumo riesgoso de alcohol (AUDIT score ≥ 8). En pacientes VIH-positivos, en el análisis multivariable síntomas depresivos (p = 0.03) y uso problemático de drogas (p = 0.007) estuvieron asociados con consumo riesgoso de alcohol. En pacientes VIH-negativos no se observaron tales asociaciones. En pacientes VIH-positivos el consumo riesgoso de alcohol es prevalente y conjuntamente con la presencia de síntomas depresivos y el uso problemático de drogas, puede influenciar la progresión de la enfermedad por VIH y el bienestar de los pacientes.

## Introduction

Risky alcohol consumption is a pattern of alcohol use that may cause physical or/and mental damage to health [1, 2]. The Alcohol Use Disorder Identification Test (AUDIT) is a validated, widely-used method of screening for levels of risky alcohol consumption [1]. Studies estimating the prevalence of risky alcohol consumption among HIV-positive individuals using the full AUDIT test [1, 3] are scarce. Previous studies estimating the prevalence of risky alcohol consumption among HIV-positive populations in the UK have commonly used a shorter AUDIT-C version (first three AUDIT questions only) [4] of the questionnaire defining individuals as risky alcohol consumers when scoring over varying thresholds [5, 6].

Among different European cohorts of HIV-positive individuals, the prevalence of risky alcohol consumption has varied. Of 111 Irish HIV-positive individuals attending an inner-city HIV clinic 31% were so called AUDIT positive, reporting risky alcohol consumption (of these 4.5% drank on hazardous levels, 19% consumed on harmful levels and 7% reported likely alcohol dependency) [7]; of 2340 French HIV positive individuals receiving highly active antiretroviral therapy (HAART) 27% scored more than 5 (> 4 for women) in the first three AUDIT questions (AUDIT-C) [8] and of 332 HIV-positive individuals attending an HIV clinic for routine care in the UK 50% scored 4 or more in the first 3 AUDIT questions indicating a risk for alcohol disorders [6]. In another study assessing recreational drug use, 65% of HIV-positive men who have sex with men (MSM) living in the UK scored 8 or more in a modified AUDIT-C questionnaire indicating harmful alcohol consumption [5].

Alcohol misuse has been associated with negative consequences in HIV-positive patients, including lower antiretroviral medication adherence [9] and accelerated HIV disease progression [10]. Previous studies have shown a relationship between increased alcohol consumption, measured as a number of drinks per day, and increased levels of self-reported anxiety and stress symptoms among HIV-positive individuals [11], as well as between alcohol use disorders and depression among HIV-negative individuals [12]. Alcohol consumption has also been associated with high risk sexual behaviours, such as unprotected sex among both populations [13, 14].

In this era of HAART, HIV-positive patients are living longer with life expectancy near or at that of the HIV-negative population. At the present time, the older cohort of HIV-positive patients is experiencing aging and its related comorbidities, such as metabolic diseases, malignancies and cardiovascular disease, whereas the younger cohort will be aging into this in the next few decades. It has been shown that co-morbidities may be experienced at a higher rate in the HIV-positive population than among HIV-negative individuals [15, 16], and that excessive alcohol use is associated with an increased risk of cardiovascular disease [17] and mortality in patients with metabolic syndromes [18]. All of which could create a population of HIV-positive patients with an increased risk of cardiovascular disease augmented further by risky alcohol consumption.

The aim of the present study was to estimate the prevalence of risky alcohol consumption, and associated health factors among HIV-positive and comparable HIV-negative patients attending a single urban HIV/sexual health clinic. To the best of our knowledge, no studies have used the full AUDIT questionnaire together with other validated standardised questionnaires of mental health, drug use and sexual behaviour to estimate the prevalence of risky alcohol consumption and associated health factors among HIV-positive individuals in the UK. Due to the lack of previous data the study had an exploratory nature. Estimating the prevalence of risky alcohol consumption in a sample of HIV-positive and comparable HIV-negative patients improves knowledge of the role of risky alcohol consumption on mental health, drug use, sexual behaviour and antiretroviral therapy (ART) adherence.

## Methods

We conducted an anonymous cross-sectional self-administered pen-and-paper questionnaire study to adult (over the age of 18 years) HIV-positive patients and comparable HIV-negative patients attending for routine care in a single urban HIV/sexual health clinic in Central London. Between 15th May 2017 and 5th January 2018 all adult patients attending the clinic were invited to complete a voluntary questionnaire comprised of the following standardised and validated instruments: the Alcohol Use Disorder Identification Test (AUDIT) to estimate risky alcohol consumption [1]; the Patient Health Questionnaire-9 (PHQ-9) to assess depressive symptoms [19], the Drug Use Disorders Identification Test (DUDIT) to screen for problematic drug use [20] and The Centre for Adherence Support Evaluation (CASE) Adherence Index to assess adherence to ART among HIV-positive participants [21]. Data on demographic characteristics, smoking status and sexual behaviour in previous 3 months were also collected.

### Data Collection

The clinic patients completed the questionnaires, with assistance from a trained clinical research assistant if requested. The participants returned the completed questionnaires to a designated mail box in the clinic waiting area. The data were entered and managed using REDCap electronic data capture tool hosted at University College London [22]. The data was analysed using STATA release 14 (StataCorp. 2015. Stata Statistical Software: Release 14. College Station, TX: StataCorp LP).

The study received a favourable opinion from the London - Bloomsbury Research Ethics Committee (Ref: 17/LO/0331). As the questionnaire was anonymous and participation voluntary a written consent was not sought; completion of the survey denoted consent. No identifiable patient data were obtained or recorded. The questionnaire included information on support services relating to alcohol and drug use, should patients feel they required any additional help.

Confirmed by month and year of birth, no patients under the age of 18 years completed the survey. The patients most commonly attend the clinic bi-annually, and therefore to avoid patients completing the questionnaire repeatedly the recruitment was ceased after 8 months.

### Demographic and Sexual Behaviour Data Collected

Demographic data collected included: gender, age (month and year of birth), employment status (employed/student or retired/not working), ethnicity (white or other) and smoking status (never/ex-smoker or smoking).

Sexual behaviour data included: gender of usual sexual partner/s (male/female/both), number of sexual partners in previous 3 months, condom-less sex in previous 3 months (yes/no), frequency of condom-less sex in previous 3 months, a diagnosis of sexually transmitted infection/s (STI) in previous 3 months (yes/no; if yes, name/s of STI/s), participation in Chemsex in previous 3 months (“used following drugs before or during sex; GHB/GBL, mephedrone, crystal meth, cocaine, ketamine and/or ‘legal highs’”) in previous 3 months and having had sex drunk in previous 3 months (“been very drunk before or during sex”).

### The Validated Questionnaire Instruments

The Alcohol Use Disorder Identification Test (AUDIT), developed by the World Health Organization (WHO), is a validated, widely-used method of screening for levels of risky alcohol consumption. The AUDIT is composed of ten items scored between 0 and 4, with higher scores indicating greater alcohol consumption and higher risk of alcohol-related problems. A total score from 0 to 40 is a sum of the individual item scores. The scores are generally converted to standard categories of sensible drinking (0–7), hazardous drinking (8–15), harmful drinking (16–19) and likely alcohol dependency if the individual scores above 20 (20–40). Hazardous drinking is when adverse consequences of alcohol consumption have been increased, harmful drinking is affecting individual’s physical and/or mental health, whereas an alcohol dependent person has given higher priority to drinking compared to other activities and is likely to experience increased tolerance and physical withdrawal upon discontinuation [1, 3]. Guided by previous research [7, 23, 24], in this study we used an outcome measure of AUDIT score 8 or more to indicate risky alcohol consumption.

The Drug Use Disorders Identification Test (DUDIT) is a parallel instrument to AUDIT for identifying individuals with problematic drug use [20]. The DUDIT is composed of 11 items, scored between 0 and 4, with a total score of 44. As previously defined, we identified men and women with problematic drug use at a cut-off score of 6 or more and 2 or more respectively [20].

The Patient Health Questionnaire-9 (PHQ-9) is composed of nine items related to the Diagnostic and Statistical Manual of Mental Disorders (DSM-IV) diagnosis criteria for major depressive disorder [19]. A score between 0 (“not at all”) and 3 (“nearly every day”) is obtained according to a frequency of difficulty experienced in each item area relating to every-day activities. A total score of 0–27 is a sum of the individual item scores. Thresholds of 5, 10, 15, and 20 represent mild, moderate, moderately severe, and severe depressive symptoms, respectively [19]. In this study we defined moderate/severe depressive symptoms as a PHQ-9 score 10 or more.

The Centre for Adherence Support Evaluation (CASE) Adherence Index is a validated composite measure of self-reported antiretroviral therapy (ART) adherence among HIV-positive patients enquiring about feelings of difficulty to take ART and missed doses of ART. The CASE Adherence Index provides a total index according to individual responses; a score of more than 10 indicates good adherence, while a total score of 10 or less is related to poor adherence [21]. These cut-off points were also utilised in this study. Only participants currently receiving ART were invited to complete the CASE Adherence Index.

All variables derived from the standardised questionnaires (AUDIT; DUDIT; PHQ-9; CASE Adherence Index) were defined as indicated in the questionnaire manuals respectively. Questions, such as those relating to Chemsex participation [25] and other sexual behaviour [26], were chosen due to their utilisation in previous studies and were defined as described in those studies.

### Statistical Analysis

The main outcome, risky alcohol consumption was defined as AUDIT score 8 or more. Following from the study aim, statistical analyses were performed independently for HIV-positive and HIV-negative patients; alcohol consumption, health and sexual behaviour were not directly compared between the groups of patients.

Descriptive statistics [n (%)] on categorical variables were used to present demographic characteristics, health factors and patterns of alcohol consumption among both samples of patients. Mean age with standard deviations are presented (Tables 1, 2, 3).

**Table 1:** Participant characteristics

Characteristics	HIV-positive	HIV-negative
	n = 227	n = 69
Gender (n = 296)		
Male	208 (91.6)	65 (94.2)
Female	19 (8.4)	2 (2.9)
Non-binary	0 (0)	2 (2.9)
Sexual orientation (n = 290)		
MSM (gay and bisexual)	193 (87.0)	62 (91.2)^a^
Heterosexual men	12 (5.4)	4 (5.9)
Heterosexual women	16 (7.2)	2 (2.9)
Gay and bisexual women	1 (0.4)	0 (0)
Age (mean, SD) (n = 296)	46.16 (10.5)	40.10 (9.9)
Ethnicity (n = 295)		
White	173 (76.5)	48 (69.6)
Other	53 (23.5)	21 (30.4)
Working status (n = 296)		
Employed or student	190 (83.7)	61 (88.4)
Retired or not working	37 (16.3)	8 (11.6)

Data are presented in n (%) unless otherwise stated. Missing data accounts for differing totals

^a^Includes two patients identifying themselves as non-binary gender reporting having sex with men

**Table 2:** Health and Sexual behaviour by HIV status

	HIV-positive	HIV-negative
	n = 227	n = 69
Health behaviour		
Depressive symptoms (PHQ-9) (n = 288)		
None/mild	195 (89.0)	60 (87.0)
Moderate/severe	24 (11.0)	9 (13.0)
Problematic drug use (DUDIT) (n = 268)		
No	148 (72.2)	43 (68.3)
Yes	57 (27.8)	20 (31.7)
Smoking status (n = 294)		
Never/ex-smoker	183 (81.3)	57 (82.6)
Smoker	42 (18.7)	12 (17.4)
Sexual behaviour		
Have sex with (n = 290)		
Men	205 (92.3)	62 (91.2)
Women	13 (5.9)	4 (5.9)
Both	4 (1.8)	2 (2.9)
Number of sexual partners (n = 283)		
None to two partners	118 (54.4)	9 (13.6)
3 or more partners	99 (45.6)	57 (86.4)
Unprotected sex (n = 254)		
No	83 (44.6)	9 (13.2)
Yes	103 (55.4)	59 (86.8)
STI diagnosis (n = 286)		
No	185 (84.9)	47 (69.1)
Yes	33 (15.1)	21 (30.9)
Chemsex participation (n = 288)		
No	167 (75.9)	37 (54.4)
Yes	53 (24.1)	31 (45.6)
Sex drunk (n = 288)		
No	191 (86.8)	46 (67.6)
Yes	29 (13.2)	22 (32.4)
Adherence to ART		
Good	190 (87.6)	–
Poor	27 (12.4)	–

Data are presented in n (%). Missing data accounts for differing totals

**Table 3:** Risky alcohol consumption by HIV status

Patterns of alcohol consumption (AUDIT score)	HIV-positive n = 227	HIV-negative n = 69
Sensible drinking (< 8)	170 (74.9)	44 (63.8)
Risky alcohol consumption (≥ 8)	57 (25.1)	25 (36.2)
*Hazardous drinking (8–15)*	41 (18.1)	20 (29.0)
*Harmful drinking (16–19)*	7 (3.1)	5 (7.2)
*Likely alcohol dependency (20–40)*	9 (3.9)	0 (0)

Data are presented in n (%). Alcohol consumption levels in italics are sub-categories of risky alcohol consumption

In the univariate analyses, Chi squared tests were performed on categorical variables to look for associations between demographic and health factors and risky alcohol consumption separately among HIV-positive and HIV-negative samples. Proportions of risky alcohol consumers [n (%)], unadjusted odds ratios together with 95% confidence intervals (CI) and p values are presented (Table 4). Logistic regression was used to assess the adjusted associations of depressive symptoms, problematic drug use, participation in Chemsex, poor adherence to ART (HIV-positive sample only), smoking status and STI diagnosis with risky alcohol consumption separately among HIV-positive and HIV-negative samples. The odds ratios were adjusted for all other variables associated with risky alcohol consumption in the univariate analyses (p ≤ 0.10). Adjusted odds ratios together with 95% confidence intervals (CI) are presented and* p* value of ≤ 0.05 was considered significant (Table 4).

**Table 4:** Health and sexual behaviour variables associated with risky alcohol consumption by HIV status: n, row  %, unadjusted and multivariate logistic regression

Health and sexual behaviour variables	Proportion of risky alcohol consumers (n = 57/227; 25.1%)	HIV-positive				Proportion of risky alcohol consumers (n = 25/69; 36.2%)	HIV-negative			
		OR (95% CI)^a^	p value	aOR (95% CI)^b^	p value		OR (95% CI)^a^	p value	aOR (95% CI)^b^	p value
Depressive symptoms (PHQ-9)^c^										
None/mild	39/195 (20.0)	1.00		1.00		19/60 (31.7)	1.00		1.00	
Moderate/severe	14/24 (58.3)	5.6 (2.23–14.09)	**< 0.001**	3.13 (1.12–8.77)	**0.03**	6/9 (66.7)	4.32 (0.92–20.27)	0.05	3.99 (0.85–18.63)	0.08
Problematic drug use (DUDIT)^d^										
No	25/148 (16.9)	1.00		1.00		12/43 (27.9)	1.00		1.00	
Yes	28/57 (49.1)	4.75 (2.32–9.72)	**< 0.001**	3.60 (1.42–9.14)	**0.007**	10/20 (50.0)	2.58 (0.83–8.1)	0.09	2.29 (0.60–8.76)	0.22
Participation in Chemsex										
No	32/167 (19.2)	1.00		1.00		12/37 (32.43)	–		–	
Yes	23/53 (43.4)	3.23 (1.63–6.43)	**< 0.001**	1.22 (0.46–3.21)	0.69	12/31 (38.71)	–		–	
Poor adherence to ART^e^										
No	42/190 (22.1)	1.00		1.00		–	–		–	
Yes	12/27 (44.4)	2.82 (1.21–6.58)	**0.01**	1.54 (0.52–4.57)	0.44	–	–		–	
Smoking status										
Never/ex-smoker	41/183 (22.4)	1.00		1.00		21/57 (36.8)	–		–	
Smoker	16/42 (38.1)	2.13 (1.04–4.39)	**0.04**	1.38 (0.57–3.34)	0.48	4/12 (33.3)	–		–	
STI diagnosis^f^										
No	42/185 (22.7)	1.00		1.00		14/47 (29.8)	–		–	
Yes	12/33 (36.4)	1.95 (0.88–4.31)	0.09	1.43 (0.55–3.73)	0.46	10/21 (47.6)	–		–	

Data are presented in n (%) unless otherwise stated. Missing data contributes to differing totals

^a^Mantel–Haenszel Test

^b^Adjusted for all other variables associated with risky alcohol consumption in univariate analyses (p < 0.10)

^c^None/mild depressive symptoms PHQ-9 score ≤ 9; Moderate/severe depressive symptoms PHQ-9 score ≥ 10

^d^Problematic drug use DUDIT score men ≥ 6 and women ≥ 2

^e^CASE Adherence Index score > 10

^f^Reporting having been given a diagnosis of any STI in previous 3 months

Those patients whose AUDIT data were missing or incomplete were excluded from the analysis (n = 35). For other instruments, the proportions of missing answers are reported. Demographic and health factors [n (%)] were compared between those patients who provided AUDIT data and those whose AUDIT data were incomplete or missing using the Chi squared test and unpaired t-test for comparing mean age. *P* value of  ≤  0.05 was considered significant (Table S1).

Proportions of problematic drug use, Chemsex participation and risky alcohol consumption was further examined among all patients between individuals who reported no Chemsex participation or problematic drug use, who reported only one of these factors, and patients who reported both Chemsex participation and problematic drug use. Proportions [n (%)] and unadjusted odd ratios together with 95% CI and p values are presented for this analysis. *P* value of ≤ 0.05 was considered significant (Table 5).

**Table 5:** Risky alcohol consumption, Chemsex participation and problematic drug use among all participants

Problematic drug use and participation in Chemsex	Proportion of risky alcohol consumers (n = 73/263; 27.8%)^a^	OR (95% CI)^b^	p value
No problematic drug use or Chemsex participation	27/159 (17.0)	1.00	–
Problematic drug use but no Chemsex participation	11/21 (52.4)	5.38 (2.08–13.92)	**0.001**
Chemsex participation but no problematic drug use	8/27 (29.6)	2.06 (0.82–5.19)	0.126
Chemsex participation and problematic drug use	27/56 (48.2)	4.55 (2.33–8.88)	**< 0.001**

^a^Of the total 296 patients 33 patients’ Chemsex and/or DUDIT data were missing

^b^Mantel–Haenszel Test

## Results

Three hundred and thirty-one patients completed and returned the questionnaire during the eight months of recruitment. The AUDIT data were incomplete or missing for 35 patients (35/331; 10.6%). Therefore, data on 227 HIV-positive and 69 HIV-negative patients were included in the analysis (Table 1). Those patients with incomplete or missing AUDIT data were more likely to be HIV-positive (p = 0.046), current smokers (p = 0.041) and have had three or more sexual partners in previous three months (p = 0.015) than patients with complete AUDIT data. Other variables were comparable (p > 0.05) (Supplementary Table I).

### Population Characteristics

Population characteristics were similar in both patient groups. Majority of HIV-positive and HIV-negative patients were MSM (87% and 91%, respectively) from white ethnic backgrounds (77% and 70%, respectively) with a mean age (SD) of 46 (11) and 40 (10), respectively, and were either in full-time employment or students (84% and 88%, respectively) (Table 1).

### Health and Sexual Behaviour by HIV Status

The patient groups reported similar health behaviours. Depressive symptoms (PHQ-9 score ≥ 10), problematic drug use (DUDIT score men ≥ 6; women ≥ 2) and smoking were reported in 11% and 13%, 28% and 32% and 19% and 17% among HIV-positive and HIV-negative patients respectively. HIV-negative patients reported more high-risk sexual behaviours compared to HIV-positive patients; they reported more often three or more sexual partners (86% vs. 46%), more often unprotected sex (87% vs. 55%), higher rates of STI diagnoses (31% vs. 15%), participation in Chemsex (46% vs. 24%) and having had sex drunk (32% vs. 13%) compared to HIV-positive patients in the three months preceding the questionnaire (Table 2).

Majority (217/227; 96%) of HIV-positive patients reported currently receiving ART, of which 95% (206/217) reported their viral load being undetectable at last test. Seven patients reported having had detectable viral loads, and four patients were unsure (data not shown). Majority (88%) of HIV-positive patients adhered well to their ART (CASE Adherence Index score > 10) [21] (Table 2).

### Risky Alcohol Consumption by HIV Status

Fifty-seven (25%) HIV-positive patients reported risky alcohol consumption, whereas 25 (36%) HIV-negative individuals reported drinking alcohol on risky levels. Among HIV-positive patients, 41 (18%) reported hazardous drinking, 7 (3%) individuals were drinking on harmful levels, and 9 (4%) participants were likely to be alcohol dependent. Among HIV-negative patients, 20 (29%) were drinking on hazardous levels and 5 (7%) patients reported harmful alcohol consumption. No potentially alcohol dependent individuals were identified among HIV-negative patients (Table 3).

### Factors Associated with Risky Alcohol Consumption by HIV Status

Among HIV-positive patients on univariate analyses there were no significant associations between risky alcohol consumption and gender, age, ethnicity, employment status, sexual orientation, number of sexual partners in the previous 3 months and unprotected sex (p > 0.10; data not shown). Presence of depressive symptoms (p < 0.001), problematic drug use (p < 0.001), Chemsex participation (p < 0.001), poor adherence to ART (p = 0.01) and smoking (p = 0.04) were associated with risky alcohol consumption among HIV-positive patients in univariate analyses. Depressive symptoms (p = 0.03) and problematic drug use (p = 0.007) remained significant in multivariate analysis after adjusting for all other variables associated with risky alcohol consumption in the univariate analysis (Table 4).

Among HIV-negative patients the presence of depressive symptoms and problematic drug use had borderline associations with risky alcohol consumption (p = 0.05 and p = 0.09, respectively) in univariate analyses. In multivariate analysis these associations diminished (Table 4).

We considered that an interaction between Chemsex participation and problematic drug use may have existed and further examined this. Among all respondents, in a comparison to those patients who did not report either problematic drug use or Chemsex participation (n = 159), those patients who reported problematic drug use only (OR 5.38, CI 2.08–13.92, p = 0.001) or those who reported both behaviours (OR 4.55, CI 2.33–8.88, p < 0.001) were more likely to be drinking on risky levels (Table 5).

## Discussion

In this study we found that among HIV-positive patients the prevalence of risky alcohol consumption was 25%, whereas among HIV-negative patients it was 36%. Similar prevalences have been reported in previous studies [7, 8, 23], but the use of varied outcome instruments and inconsistent definitions of risky alcohol consumption have made the comparison of findings between these studies challenging [27].

According to WHO, the total population (aged > 15 years) prevalence of alcohol use disorders in the UK in 2016 was 8.7%, whereas 1.4% of the adult British population were alcohol dependent [2]. A study assessing the effectiveness of screening and brief alcohol intervention in primary care settings in the UK found that 30.1% of 2991 patients reported risky alcohol consumption using the AUDIT questionnaire [23]. Among HIV-positive patients, Michel et al. (2010) found that 27% of 2340 French HIV-positive individuals reported harmful alcohol consumption using the shorter AUDIT-C questionnaire [8], whereas in the UK, half (50%) of HIV-positive patients attending an urban HIV clinic for routine care scored 4 or more in the AUDIT-C questionnaire indicating harmful alcohol consumption [6]. By using the full AUDIT questionnaire, our study has provided useful novel data on patterns of alcohol consumption among HIV-positive patients and HIV-negative population.

In our study noticeable was that even though HIV-negative patients reported a higher prevalence of risky alcohol consumption, the pattern of alcohol use among HIV-positive patients was of higher risk; 4% of HIV-positive patients were likely to be alcohol dependent, whereas none of the HIV-negative patients reported likely alcohol dependency. High levels of risky drinking among HIV-positive patients have been reported previously. Surah et al. (2013) found a 31% prevalence of risky alcohol consumption (AUDIT score ≥ 8) in an Irish HIV-positive inner-city population; further examining the patterns of risky alcohol consumption 4.5% of their participants reported hazardous drinking, 19% were harmful drinkers and 7% reported likely alcohol dependency [7]. The corresponding figures in our study were 18.1%; 3.1% and 3.9% respectively. Gonzalez Baeza et al. reported a pattern of higher levels of risky and hazardous drinking, but lower levels of likely alcohol dependency among British patients participating in a study assessing cognitive performance among HIV-positive individuals; 36.3% of their participants reported risky alcohol consumption (AUDIT score ≥ 8), 29.5% were drinking on hazardous levels, 6.2% reported harmful alcohol consumption and 0.7% had likely alcohol dependency [28].

In the multivariable analysis among HIV-positive patients only moderate or severe depressive symptoms and problematic drug use were associated with risky alcohol consumption. These associations were not detected among HIV-negative patients in the multivariable analysis. Eleven percent of HIV-positive patients reported moderate or severe depressive symptoms when measured using the PHQ-9 questionnaire. Previous studies utilising the PHQ-9 questionnaire have reported higher prevalences of depression among HIV-positive patients compared to our study, ranging from 14.5 to 27% [29–31]. Sociodemographic characteristics of our patients may in part explain the disparities in findings. Increased depressive symptoms among HIV-positive patients have been associated with female gender, low socio-economic status and younger age [30, 31], whereas majority of our patients were men, in full-time employment or students and had a mean age of 46 years. However, the association between moderate or severe depressive symptoms and risky alcohol consumption found in this study highlights the importance to screen for lower levels of alcohol consumption among HIV-positive patients, as the majority of our patients who reported risky alcohol consumption were consuming on hazardous levels.

The association between increased alcohol consumption and depressive symptoms is well established in research among the general population [12], whereas among HIV-positive patients, even though increased alcohol consumption and mood disorder are both prevalent, they are less often studied together [27]. Although our study’s cross-sectional design prevents us from examining the potential causal relationship between depressive symptoms and risky alcohol consumption among HIV-positive individuals, previous studies have highlighted factors such as psychosocial burden relating to having a life-long illness and experiences of social stigma, which may lead to risk behaviours such as increased alcohol consumption [32]. Co-occurrence of risky alcohol consumption and depression may affect adherence to ART, HIV disease progression, as well as the individual’s experience of living with HIV. Studies assessing substance use and depressive symptoms among HIV-positive patients have commonly focused on drug use [27].

Almost a quarter (24.1%) of HIV-positive patients and 45.6% of HIV-negative patients reported having participated in Chemsex in previous 3 months. As a comparison, a previous study on Chemsex participation among HIV-positive individuals reported a prevalence of 29.5%, and found no association between binge drinking and sexualized drug use [25]. Among HIV-positive patients in our study, Chemsex participation was associated with risky alcohol consumption in the univariate analysis, but after adjusting for other variables associated with risky alcohol consumption in the multivariate analysis, the significance of Chemsex participation diminished. In contrast, problematic drug use remained significant in the multivariate analysis.

Pufall et al. reported an independent association between Chemsex participation and recreational drug use, whereas frequency of binge drinking was not associated with Chemsex participation in their sample [25]. Similarly, in our study among all patients the association with risky alcohol consumption was strongest among those patients who reported problematic drug use only and Chemsex participation together with problematic drug use. Important is to note that in our study Chemsex participation and problematic drug use were not entirely independent variables; if the patient had participated in Chemsex in the previous 3 months and therefore reported using drugs, they were more likely to score above the defined threshold of problematic drug use in the DUDIT questionnaire. We are unable to determine whether those patients who reported Chemsex participation and problematic drug use also used drugs outside the Chemsex context, but rather that these two variables were associated. Small stratum numbers (n < 10) need to be noted in the interpretation of these results.

An advantage to our study was that the questionnaire was composed of standardised and validated instruments supporting repeatability and allowing direct comparisons to previous studies utilising the same instruments among both HIV-positive and HIV-negative populations. In addition, we aimed to recruit individuals with and without HIV-infection in order to estimate the prevalence of risky alcohol consumption and other health factors among both patient populations. The HIV-negative comparison group was recruited from associated sexual health clinic, they had similar demographic characteristics to that of the HIV-positive individuals, reported high risk sexual behaviours and therefore were at increased risk of acquiring the infection. Thus, a more comparable patient population than a general population sample would have yielded.

As a limitation, due to the nature of the recruitment strategy we were unable to record sociodemographic or general information of the clinic attendees who declined to complete the survey, and the study sample was self-selected. When attending the clinic those patients with increased alcohol or drug use, or depressive symptoms might have been less willing to take part in additional activities not directly related to their routine care, and therefore less likely to contribute data. This might in part explain the lower levels of depressive symptoms reported by our patients compared to other similar populations, as discussed above. In addition, a patient might have attended the clinic more than once during the 8 months of recruitment and therefore be invited to complete the questionnaire repeatedly. However, as the recruitment period was relatively short it was considered unlikely that patients did not recall already participating or were willing to participate repeatedly.

As a conclusion, this study has provided important novel data on the prevalence of risky alcohol consumption and associated health factors among HIV-positive and comparable HIV-negative patients using standardised and validated instruments. A quarter (25.1%) of our HIV-positive patients and 36.2% of HIV-negative patients reported risky alcohol consumption. Due to the lack of previous data, this study had an exploratory nature. Our findings highlight the need for further research, especially on associations between and impact of co-occurring risky alcohol consumption, depressive symptoms and problematic drug use among HIV-positive individuals.

## Electronic supplementary material

Below is the link to the electronic supplementary material.

Supplementary material 1 (DOCX 21 kb)
