# Supplementary material for: Risky Alcohol Consumption and Associated Health Behaviour Among HIV-Positive and HIV-Negative Patients in a UK Sexual Health and HIV Clinic: A Cross-Sectional Questionnaire Study
Source: AIDS Behav. 2019 Oct 29;24(6):1717–26. doi: 10.1007/s10461-019-02714-2 (PMC7220979; doi:10.1007/s10461-019-02714-2)
Supplement: Supplementary file 1 — Supplementary material 1 (DOCX 21 kb) [file 10461_2019_2714_MOESM1_ESM.docx]

Supplementary Table I: A Comparison of Patient Characteristics, Sexual and Health Behaviour among participants with complete and missing AUDIT data

|  | **AUDIT data available** | **Missing** | **AUDIT data missing** | **Missing** | **p-value^a^** |
| --- | --- | --- | --- | --- | --- |
|  | **n=296 (89.4)** |  | **n=35 (10.6)** |  |  |
| **Patient characteristics** | |  |  |  |  |
| HIV status | | 0 |  | 0 |  |
| Negative | 69 (23.31) |  | 3 (8.57) |  |  |
| Positive | 227 (76.69) |  | 32 (91.43) |  | **0.046** |
| Male | 273 (92.23) | 0 | 30 (85.71) | 1 (2.86) | 0.560 |
| Age (mean, SD) | 44.7 (10.66) | 8 (2.70) | 47.94 (7.83) | 3 (8.57) | 0.097^b^ |
| Ethnicity |  | 1 (0.34) |  | 1 (2.86) |  |
| White | 221 (74.66) |  | 23 (65.71) |  |  |
| Other | 74 (25.00) |  | 11 (31.43) |  | 0.359 |
| Working status | | 0 |  | 1 (2.86) |  |
| Employed or student | 251 (84.80) |  | 28 (80.00) |  |  |
| Retired or not working | 45 (15.20) |  | 6 (17.14) |  | 0.709 |
| **Health behaviour** | |  |  |  |  |
| Depressive symptoms (PHQ-9) | | 8 (2.70) |  | 9 (25.71) |  |
| None/mild | 255 (86.15) |  | 22 (62.86) |  |  |
| Moderate/severe | 33 (11.15) |  | 4 (11.43) |  | 0.552 |
| Smoking status | | 2 (0.68) |  | 2 (5.71) |  |
| Never/ex-smoker | 240 (81.08) |  | 22 (62.86) |  |  |
| Smoker | 54 (18.24) |  | 11 (31.43) |  | **0.041** |
| Problematic drug use (DUDIT) | | 28 (9.46) |  | 25 (71.43) |  |
| No | 191 (64.53) |  | 7 (20.00) |  |  |
| Yes | 77 (26.01) |  | 3 (8.57) |  | 0.931 |
| Adherence to ART^c^ | |  |  |  |  |
| Good | 192 (87.67) |  | 24 (96.00) |  |  |
| Poor | 27 (12.33) |  | 1 (4.00) |  | 0.216 |
| **Sexual behaviour** | |  |  |  |  |
| Have sex with | | 6 (2.03) |  | 7 (20.00) |  |
| Men | 267 (90.20) |  | 26 (74.29) |  |  |
| Women | 17 (5.74) |  | 2 (5.71) |  |  |
| Both | 6 (2.03) |  | 0 (0.00) |  | 0.722 |
| Number of sexual partners in last 3 months | | 13 (4.39) |  | 16 (45.71) |  |
| 0 to 2 partners | 127 (42.91) |  | 14 (40.00) |  |  |
| 3 or more partners | 156 (52.70) |  | 5 (14.29) |  | **0.015** |
| Unprotected sex in last 3 months | | 42 (14.12) |  | 16 (45.71) |  |
| No | 92 (31.08) |  | 9 (25.71) |  |  |
| Yes | 162 (54.73) |  | 10 (28.57) |  | 0.332 |
| STI diagnosis in the last 3 months | | 10 (3.38) |  | 12 (34.29) |  |
| No | 232 (78.38) |  | 21 (60.00) |  |  |
| Yes | 54 (18.24) |  | 2 (5.71) |  | 0.222 |
| Chemsex participation in the last 3 months | | 8 (2.70) |  | 9 (25.71) |  |
| No | 204 (68.92) |  | 22 (62.86) |  |  |
| Yes | 84 (28.38) |  | 4 (11.43) |  | 0.134 |
| Sex drunk in the last 3 months | | 8 (2.70) |  | 10 (28.57) |  |
| No | 237 (80.07) |  | 24 (68.57) |  |  |
| Yes | 51 (17.23) |  | 1 (2.86) |  | 0.077 |
| Data are presented in n (%) unless otherwise stated  ^a^ Chi Squared test  ^b^ Unpaired t-test  ^c^ Among those patients who provided data | | | | | |
